# Frequency and Determinants of Inappropriate Use of Treadmill Stress Test for Coronary Artery Disease

**DOI:** 10.7759/cureus.2101

**Published:** 2018-01-23

**Authors:** Muhammad Bilal, Abdul Haseeb, Mohammad H Arshad, Altamash A Jaliawala, Iman Farooqui, Amna Minhas, Ahmedullah Hussaini, Arsalan A Khan, Sharjeel Ahmad, Zainab Saleem, Ozair Awan, Noor Us Sabahat, Araib Ayaz, Haania Rizwan

**Affiliations:** 1 Department of Medicine, Dow University of Health Sciences (DUHS), Karachi, Pakistan; 2 Internal Medicine, Aga Khan University Hospital; 3 Department of Biology, Karachi Grammar School; 4 Science, Karachi Grammar School; 5 Biology Department, Karachi Grammar School; 6 Biology, The Lyceum School, Karachi; 7 Student, Dow University of Health Sciences (DUHS), Karachi, Pakistan; 8 Biology, Karachi Grammar School; 9 Department of Medicine, The Lyceum School, Karachi; 10 St.michael’s Convent School

**Keywords:** exercise tolerance test, treadmill test, coronary artery disease, healthcare system

## Abstract

Background

In developing countries like Pakistan, treatment is mediated by private and public healthcare setups with a limited budget for health facilities. Moreover, the inappropriate use of treadmill tests imposes a burden on healthcare resources and leads to unwarranted interventions. Our aim is to assess the prevalence and predictors of inappropriate referrals for the exercise tolerance test (ETT) to diagnose coronary artery disease (CAD) while taking public and private healthcare settings into consideration.

Methods

A cross-sectional study was conducted to find the prevalence of the inappropriate use of ETT to diagnose obstructive CAD and to determine the factors responsible for it. A total of 264 patients were enrolled from outpatient departments in Karachi. The inclusion criterion was the referral of treadmill testing for the diagnosis of CAT. The analysis was performed by logistic regression models to ascertain independent predictors of inappropriate use.

Results

Exercise stress tests were found to be inappropriate in 209 (79%) patients. The study indicated that the majority of patients had a low or very low pre-test probability of CAD. Diabetes, hypertension, and dyslipidemia were less frequent in the inappropriate as compared to the appropriate referrals (10%, 45%, and 16% versus 20%, 69%, and 32%). Both public and private sectors showed a high prevalence of inappropriate testing, but it was much higher in the latter (27% versus 73%, P < 0.001). In all regression models, the private healthcare system was the major independent predictor for inappropriate indications of ETT with an average odds ratio of 4.9 (P < 0.001).

Conclusion

The high prevalence of ETT referrals was found for the diagnosis of CAD. This result was consistent with both public and private healthcare systems, but it was considerably higher in private setups. Comorbidities, number of risk factors, and cardiovascular risk were not associated with the inappropriate use of ETT.

## Introduction

The exercise treadmill stress test, a noninvasive diagnostic test, is performed for the identification and prompt management of coronary artery disease (CAD) [1]. Since it is easy to perform, safe, and relatively cheaper than other investigations, such as nuclear imaging, echocardiography, and coronary angiography, it has become the most common modality to assess CAD [2]. However, literature has failed to reveal that increased screening for CAD improves cardiovascular disease in primary prevention approaches. Additionally, diagnosing ischemic response in asymptomatic patients with CAD has also failed to yield beneficial results [3]. Since it is easily available and affordable by the masses, it is susceptible to overuse in healthcare setups. It is anticipated that this overuse may yield over-diagnosis and over-treatment of CAD [4-5].

Developing countries, such as Pakistan, where treatment is mediated by private and public healthcare setups, have limited and fixed budgets for health facilities. On the other hand, in developed countries, such as France and Germany, with universal healthcare systems, resources allocated for health are five times greater than in developing countries [6]. The incorrect usage of medical tests and procedures needs to be discouraged because they are an unnecessary and preventable burden on resources that could be better utilized elsewhere. Consequently, the increasing expenses on healthcare facilities in developed and developing countries have become a prime concern these days. It has been estimated that 20% of health spending in the US represents an unnecessary drainage of healthcare resources, which is mainly composed of the inappropriate use of diagnostic evaluations [7].

Very few studies have been conducted to determine the incorrect use of the treadmill test and local literature on this issue remains limited. Therefore, this study was aimed to evaluate the prevalence and predictors of the inappropriate use of the treadmill stress test for CAD in healthcare setups of Pakistan.

## Materials and methods

A cross-sectional study was conducted from June 2015 to September 2015 on 264 participants, who were referred to undergo the treadmill exercise stress test for the assessment of CAD from outpatient departments (OPDs) in Karachi. The subjects were clinically evaluated by cardiologists having a minimum experience of five years, to identify the symptoms and risk factors of CAD. We employed the Diamond-Forrester algorithm to evaluate pre-test probability for CAD [8]. The algorithm consisted of age, sex, and chest pain characteristics. Furthermore, the cardiovascular (CV) risk was determined by considering the risk prognosis guidelines of World Health Organization (WHO), which consisted of age, sex, blood pressure (BP), diabetes, and smoking status of the subject [9]. Consequently, participants were then stratified into three categories. A low risk was determined if CV risk was approximated to be < 10 % in 10 years, a risk of intermediate degree if it ranged between 10% and 20%, and lastly, a high risk was taken into account if it exceeded 20%.

A previous Brazilian study reported 78% prevalence of inappropriate referrals of the treadmill tests. Using this prevalence, at a confidence level of 95%, a sample size of 264 was calculated from the open EPI calculator. The research protocol was approved by the Institutional Review Board (IRB) of Dow University of Health Sciences.

To distinguish an inappropriate referral, we used the definition proposed by Silva et al. [10]. According to this definition, if the patient presented with one of the following conditions, he was considered as an inappropriate referral. First, subjects asymptomatic for CAD and, second, symptomatic subjects with a low or high pretest probability of CAD. Moreover, referrals in symptomatic subjects with known CAD and participants of age above 70 years presenting chest pain were categorized as appropriate. For subjects younger than 30 years of age, we classified them with a probability of CAD according to an age interval of 30-39 years in the criteria of Diamond-Forrester.

The occurrence of an inappropriate referral was determined by the proportion method, and its accuracy was described at a confidence interval of 95%. During a univariate analysis, appropriate variables were compared with inappropriate referrals by employing chi-square test for categorical variables and the student t-test was used for numerical data. A P value of < 0.05 was considered significant.

Data entry and analysis was performed using SPSS software (version 19.0, SPSS Inc., Chicago, USA). The independent predictors of inappropriate referrals were determined by inserting the variables of a univariate analysis with P < 0.10 in the multivariate logistic regression models by applying a backward technique. Consequently, three logistic regression models were employed to depict the correlating parameters obtained from the univariate analysis. Moreover, these regression models were also correlated to clinical thinking in the diagnostic evaluation request. Besides, model 1 was adjusted for clinical variables, such as hypertension (HTN), diabetes, and dyslipidemia; the second model was adjusted for the number of risk factors in an individual, and the third model was correlated with cardiovascular risk in the patients referred for the treadmill test. These correlations were later illustrated by odds ratios and 95% confidence interval. The variables that were present in the final model with a P value of less than < 0.05 were designated as independent predictors. Furthermore, the calibration of the models was gauged by using the Hosmer-Lemeshow test.

## Results

A total of 264 subjects who underwent the treadmill exercise test and met inclusion criteria were enrolled in the research during the aforementioned time period. The mean age of study subjects was found to be 51±13 years. Our study consisted of 54.2% (N=143) females and 53.6% (N=140) asymptomatic participants. Our results indicated a preponderance of participants having a low or very low (26.1% and 45.8%, respectively) pre-test probability for CAD and a low cardiovascular risk (81.8%), in accordance with the WHO risk prediction. Moreover, 66% percent of the diagnostic evaluations in the sample were obtained from the private healthcare system. The other sociodemographic features and clinical history of the study participants are depicted in Table 1.

**Table 1:** Demographic features of study subjects ^1^Symptom in the anamnesis; ^2^Age ≥ 70 years or known CAD; ^3^Cardiovascular risk estimation in 10 years, according to the WHO risk prediction (Low: <10%, Intermediate: between 10% and 20%, High: ≥ 20%)

Parameters	Frequency (%)
N	264
Age (±years)	51±13
Gender	
Male	121 (45.8)
Symptom of chest pain^1^	
Asymptomatic	140 (53.6%)
Nonanginal	105 (39.8%)
Atypical angina	12 (4.5%)
Typical angina	7 (2.7%)
Pre-test probability of CAD	
Very low	121 (45.8%)
Low	69 (26.1%)
Intermediate	42 (15.9%)
High	2 (0.8%)
Not applicable^ 2^	30 (11.4%)
Hypertension	132 (50.0%)
Diabetes	32 (12.1%)
Dyslipidemia	53 (20.1)
Obesity	70 (26.5%)
Family history of CAD	61 (23.1%)
Smoker	21 (8.0%)
Known CAD	18 (6.8%)
Previous myocardial infarction	14 (5.3%)
Revascularization	16 (6.1%)
Cardiovascular risk ^3^	
Low	216 (81.8%)
Intermediate	24 (9.1%)
High	24 (9.1%)
Tests of public health system	90 (34.1%)

Among 264 subjects, the diagnostic evaluations in our study assessed 79% (N=209) of subjects who underwent the treadmill exercise stress test to be inappropriate. The frequencies of the inappropriate treadmill stress test were observed to be higher in the private healthcare setup than in the public health care system.

Additionally, subjects who underwent appropriate tests were significantly older than those who underwent inappropriate tests (58±13 years vs. 42±14 years, P <0.001). The frequency of risk factors like diabetes, HTN, and dyslipidemia was lower in participants who were assessed with inappropriate referrals, manifesting their low cardiovascular risk profiles/status. The rest of the clinical characteristics of the participants with appropriate and inappropriate referrals were found to be insignificant (P value > 0.05) and are depicted in Table 2.

**Table 2:** Patient characteristics according to exercise stress test appropriateness criteria ^a^Number of risk factors (hypertension, diabetes, dyslipidemia, obesity, family history of CAD, smoking); ^b^Cardiovascular risk estimation in 10 years, according to the WHO risk prediction (Low: <10%, Intermediate: between 10% and 20%, High: ≥ 20%)

Parameter	Appropriate	Inappropriate	P-value
	N=55	N=209	
Males	29(52.7%)	92(44.0%)	0.22
Age	58±13	42±14	<0.001
Clinical conditions			
Diabetes	11 (20.0%)	21 (10.0%)	0.04
Dyslipidemia	18 (32.7%)	35 (16.7%)	0.05
Hypertension	38 (69.1%)	94 (45.0%)	<0.001
Obesity	17 (31.0%)	53 (25.4%)	0.43
Previous infarction	3 (5.5%)	11 (5.3%)	0.61
Known CAD	6 (10.9%)	12 (5.7%)	0.33
Revascularization	6 (10.9%)	10 (4.8%)	0.28
Smoker	2 (3.6%)	19 (9.1%)	0.21
Family history of CAD	14 (25.5%)	47 (22.5%)	
Risk factors^a^			0.02
No risk factor	8 (14.5%)	75 (35.9%)	
1 or 2 risk factors	33 (60.0%)	108 (51.7%)	
>2 risk factors	13 (23.6%)	27 (12.9%)	
Cardiovascular risk^b^			0.001
Low	33 (60.0%)	183 (87.6%)	
Intermediate	14 (25.5%)	10 (4.8%)	
High	9 (16.4%)	15 (7.2%)	
Public health system	33 (60.0%)	57 (27.3%)	<0.001

Finally, three logistic regression models were employed to illustrate the correlated variables obtained from the univariate analysis, as shown in Table 3. We excluded the age variable from these models since it was considered in the evaluation of pre-test probability (in accordance with the definition of inappropriate use of the treadmill exercise stress test). HTN was the only clinical condition that was indirectly related to the inappropriate use of the treadmill test in Model 1. Moreover, Model 2 and Model 3 identified no significant correlation between cardiovascular risk and number of risk factors with inappropriate treadmill test referrals. Later in the second stage, age was adjusted in Model 1 to determine the indirect correlation of HTN in inappropriate referrals with patients of a younger age. However, HTN turned to be insignificant in this model, while other networks of health were determined to be greatly linked (with adjustment of age, OR=3.7; P=0.004). As a result, only patients obtained from private health care remained a significant covariate in the models, with OR=4.6 (P<0.001) in accordance with the model, adapted for clinical conditions. Furthermore, the P value remained > 0.05 for all models in the Hosmer-Lemeshow test, which indicates satisfactory calibration.

**Table 3:** Determinants in multivariable logistic regression for inappropriate use of exercise stress test for coronary artery disease

	Model 1		Model 2		Model 3	
Predictors	OR (95% IC)	P Value	OR (95% IC)	P Value	OR (95% IC)	P Value
Private health system	4.6 (1.9-9.4)	<0.001	5.3 (2.8-10.3)	<0.001	4.9 (2.6-10.7)	<0.001
Clinical conditions	-	-	Not selected	-	Not selected	-
Diabetes	0.37 (0.15-1.3)	0.300	-		-	-
Dyslipidemia	0.70 (0.35-2.0)	0.511	-		-	-
Hypertension	0.40 (0.15-0.79)	0.017	-		-	-
Obesity	0.49 (0.19-1.1)	0.198	-		-	-
Risk factors (reference: without risk factor)	Not selected	-	1.00	-	Not selected	-
1 or 2 risk factors	-	-	3.4 (1.02-10.9)	0.060	-	-
>2 risk factors	-	-	1.1 (0.36-3.4)	0.490	-	-
Cardiovascular risk (reference low risk)	Not selected	-	Not selected	-	1.00	-
Intermediate	-	-	-	-	2.1 (0.59-6.1)	0.312
High	-	-	-	-	0.33 (0.07-1.8)	0.182

## Discussion

ETT has demonstrated a pivotal role in the diagnosis of CAD in the last hundred years. In this investigation, we assessed the unnecessary ETT referrals for the diagnostic workup of CAD in the healthcare setups of Pakistan. Notably, the inappropriate referrals made from private healthcare setups were five times greater than from physicians from public healthcare setups. It was also revealed that the patients' clinical condition only played a minor role in ETT recommendation.

We further identified that the majority of recommendations for ETT were inappropriate, with a prevalence of 79%. The results are consistent with similar studies conducted in other parts of the world. An Italian research conducted on 960 patients highlighted that only 33% of referrals involving non-invasive diagnostic modalities for cardiac conditions were appropriate. However, with respect to ETT, only 27% of recommendations were appropriate [11]. Another identical study conducted in Brazil is also in line with our results, indicating a prevalence of 71% [10]. The current literature on deducing the appropriateness criteria of ETT among obstructive CAD patients is inadequate. In spite of this fact, a few studies evaluating the outcomes of a screening test in asymptomatic patients with obstructive CAD revealed that they have no significant role in the diagnostic workup [12-13]. Furthermore, various investigations identified that regular myocardial stress-testing modalities in patients with diagnosed coronary artery disease were not helpful in reducing the chief CV complications [14-15]. The main efficacy of ETT to diagnose obstructive CAD remains in symptomatic patients with intermediate pretest probability [10]. A Canadian study highlighted that 42% of patients with suspected CAD and positive ETT had normal angiography results [16].

Although we discovered excessive inappropriate ETT from the public healthcare system, inappropriate diagnostic workup was significantly higher from the private healthcare setup. Hypertension was the most common comorbidity among the appropriate ETT referrals, and this outcome was in line with that described in an Italian study by Orsini et al. [17]. The multivariate models demonstrated that only being from a private health setup was an independent predictor of the inappropriate use of ETT. Surprisingly, both the CV risk and the presence of comorbidities became statistically insignificant after adjusting for the healthcare system. These findings were in congruence with the study performed in Brazil [10].

ETT is the least expensive of all non-invasive techniques used for CAD screening. However, because of its low sensitivity and specificity, an appropriate rationale must be followed to avoid unnecessary testing [18]. The irrational use of complementary diagnostic methods directly affects the healthcare expenditure. A study performed in Pakistan suggested that one in every five adults in urban parts of the country have CAD [19]. It stays a significant and emergent problem in most developing parts of the world. The rise in prevalence and mortality related to the large burden of CAD is a reflection of the epidemiological transition that has accompanied economic and social development. This transition, in many cases, outpaces the development of human resources to manage such a significant chronic disease. Therefore, a rationale needs to be developed to avoid an unnecessary burden on health care and use this important screening and diagnostic method judiciously. Moreover, a negative treadmill test may affect the screening strategies, as the false negative results may prevent the predisposed individuals from avoiding their modifiable risk factors and overall risk profile.

The results obtained from this study have few limitations with reference to generalization. The small sample size from a confined area more likely represents the local reality of a few healthcare setups in South Pakistan. Further studies and meta-analyses need to assess the different populations and widen the discussion on this pertinent subject.

## Conclusions

This study found a high prevalence of inappropriate referrals of ETT for the diagnosis of CAD. The finding was true for both public and private healthcare systems, but considerably higher in private setups. The presence of CAD risk factors, comorbidities like hypertension and diabetes, and the assessment of CV risk were not related to the inappropriate use of the treadmill test to diagnose obstructive CAD. Furthermore, the private healthcare setup was the only independent predictor of inappropriate referral. In fact, one of the most important factors for referral was the availability of modality in the setup. The results of this study may support the reasonable use of healthcare resources even when easily accessible, directing the best recommendations for the appropriate use of this important non-invasive technique.
